# Antimicrobial Activity of Six Essential Oils Against a Group of Human Pathogens: A Comparative Study

**DOI:** 10.3390/pathogens8010015

**Published:** 2019-01-28

**Authors:** Adrian Man, Luigi Santacroce, Romeo Jacob, Anca Mare, Lidia Man

**Affiliations:** 1 Department of Microbiology, University of Medicine, Pharmacy, Sciences and Technology of Târgu Mureș, Târgu Mureș 540139, Romania; mareanca@gmail.com; 2 Ionian Dept. (DJSGEM), Microbiology and Virology Lab., University Hospital of Bari, 70124 Bari, Italy; 3 Polypheno srl Academic Spin Off, University of Bari, 70124 Bari, Italy; 4 University of Medicine, Pharmacy, Sciences and Technology of Târgu Mureș, Târgu Mureș 540139, Romania; romeofloris@gmail.com; 5 Pediatrics Clinic 1, University of Medicine, Pharmacy, Sciences and Technology of Târgu Mureș, Târgu Mureș 540139, Romania; lidia.man@gmail.com

**Keywords:** antimicrobial agents, micelles, minimum inhibitory concentration (MIC), methicillin-resistant *Staphylococcus aureus* (mrsa), volatile oils

## Abstract

Essential oils are concentrated natural extracts derived from plants, which were proved to be good sources of bioactive compounds with antioxidative and antimicrobial properties. This study followed the effect of some commonly used essential oils in micellar and aqueous extract on some of the most common pathogenic bacteria. Frankincense, myrtle, thyme, lemon, oregano and lavender essential oils were tested against *Staphylococcus aureus*, *Enterococcus faecalis*, *Escherichia coli*, *Klebsiella pneumoniae* and *Pseudomonas aeruginosa*. Both micellar and aqueous extracts were used for determination of their minimal inhibitory (MIC) and bactericidal concentrations (MBC). The most active oils were oregano, thyme, lemon and lavender, while the least active were frankincense and myrtle. Oregano oil presented up to 64 times lower MICs/MBCs than ethylic alcohol, if considered as standard, on all bacteria. Most susceptible bacteria were the Gram-positive cocci, including methicillin resistant *S. aureus*, while the most resistant was *P. aeruginosa*. With some exceptions, the best activity was achieved by micelles suspension of essential oils, with MICs and MBCs ranging from 0.1% to > 50% v/v. Only oregano and lavender aqueous extracts presented bactericidal activity and only on *K. pneumoniae* (MIC = 6.3%). Thyme, lemon and oregano oils present significantly lower overall average MICs for their micellar form than for their aqueous extracts. The present results may suggest some formulas of colloid or micelle suspensions of whole essential oils such as oregano, thyme or lemon oil, that may help in antimicrobial fight. Aqueous extracts of oregano or thyme oil with good antibacterial activity could also be used in selected cases.

## 1. Introduction

Essential oils, also called volatile oils, are concentrated natural extracts derived from plants, which have been used as alternative medicines since the late twelfth century, and became more widespread in the second half of the sixteenth century. Modern chemistry allowed a deeper approach of essential oils that resulted in many publications during the nineteenth century. The essential oils started to be gradually used in the production of perfumes, foodstuffs or beverages [1]. Essential oils were proved to be good sources of bioactive compounds, with antioxidative and antimicrobial properties. Many plant parts contain oils that can be extracted: leaves, seeds, bark, resin, berries, flowers, roots or fruits. The essential oils can be purified by steam distillation, hydrodistillation, hydrodiffusion or by solvent extraction [2]. The composition is complex and consists mostly of terpenes (mostly monoterpenes and sesquiterpenes), terpenoids (oxygenated compounds such as phenols, alcohols, aldehydes, ketones or ethers) and aromatic compounds [2]. Parts of these compounds are hydrophobic, but some parts are hydrosoluble. Even though terpenes are considered hydrophobic, they may present some water solubility depending on their structure and mixing temperature. Terpenoids present better solubility in water than terpenes [3]. 

Essential oils were already successfully used in treatment of several conditions such as releasing of pain associated with chronical conditions or with medical procedures [4], reducing postoperative nausea or autonomic response to pain [5,6,7] for possible symptom relief in people with cancer [8,9] and even to treat pediculosis in children [10]. Many studies were performed on pediatric patients and no side effects were described.

Antibacterial activity of essential oils presents an increasing interest in the last years and were shown to be effective even on multidrug resistant strains [11,12,13]. In this paper, we also show a good effect of some essential oils on methicillin-resistant *Staphylococcus aureus*. Antifungal and antiviral effects were also described in the literature for some essential oils [12,14,15].

Many studies in the last years focused on the benefic properties of the essential oils, including antibacterial properties. The diversity of products, of the manufacturing processes, the high microbial diversity made possible the publication of hundreds of results, and nevertheless, this research field is widely open. This study was focused on the effect of some commonly used essential oils in our area on some of the most pathogenic bacteria, which were proved to be involved in both community-acquired and hospital-acquired infections [10,14,16].

## 2. Materials and Methods

### 2.1. Essential Oils

Six of the most commonly used essential oils in our geographic area were tested for their antibacterial properties. Frankincense (resin from *Boswellia sacra*), myrtle (*Myrtus communis*), thyme (*Thymus vulgaris*), lemon (*Citrus limon*), oregano (*Origanum vulgare*) and lavender (*Lavandula angustifolia*) essential oils were acquired from specialty retailer shops. We followed to get good quality, natural oils, with no added synthetic compounds.

### 2.2. Bacterial Strains

Six Gram-positive and Gram-negative bacteria were tested for their susceptibility to the essential oils. The chosen strains are representative for the most commonly involved pathogenic bacteria in human infections, including strains with high resistance to antibiotics or high environmental or antibiotic adaptability. Methicillin sensitive *Staphylococcus aureus*—MSSA ATCC 29213, methicillin resistant *Staphylococcus aureus*—MRSA ATCC 43300, *Enterococcus faecalis* ATCC 29212, *Escherichia coli* ATCC 25922, *Klebsiella pneumoniae* ATCC 13883, *Pseudomonas aeruginosa* ATCC 27853 were used in this study. These bacterial strains are part of the collection of the Department of Microbiology, University of Medicine, Pharmacy, Sciences and Technology of Târgu Mureș, Romania. All bacterial strains were maintained in Tryptic Soy Broth (TSB) medium with glycerin, at −70 °C. 

### 2.3. Preparation of Working Solutions

As the essential oils are hydrophobic, we used two adapted broth microdilution methods, based on CLSI 2018 standard methodology (document M07) and on previous similar studies [16,17]. 

The aim of the first method was to obtain homogenous micelle aggregates composed of water and essential oil that could be mixed with the water-based liquid culture medium. For this, 2 mL of sterile water and essential oil were mixed in in microcentrifuge tubes and the micelles were obtained by sonication at 40 kHz, for 20 min at 25 °C, in a sonicator water bath (Elmasonic S30, Elma Schmidbauer GmbH, Germany) using sweep mode. The bottom homogenous opalescent phase was carefully recovered using fine sterile pipette tips and further used as stock micelle solution of essential oil (MiEO).

The second method followed the antimicrobial activity of hydrosoluble components of the essential oils. For this, 2 mL of each pure essential oil were mixed with an equal amount of sterile water in 15 mL sterile centrifuge tubes and gently but thoroughly mixed overnight at 25 °C using an orbital plate mixer. The tubes were then centrifuged for 15 min at 5000 rpm in order to achieve a better separation of the aqueous and nonaqueous phases. After checking the complete separation, the bottom aqueous phase was recovered and further used as the second working solution of essential oil (AqEO).

### 2.4. HPLC Analysis

HPLC analysis was performed in order to assess and quantify the chemical content of the essential oils. The whole dataset includes a lot of substances extracted from both the plants used for this study and from other that we are currently testing in vitro and in vivo. All the obtained chromatogram have been matched with the main libraries of chemical compounds to confirm the type of molecules detected, mainly terpens and terpenoids. Quantitative data were also obtained.

### 2.5. Determination of the Minimum Inhibitory Concentrations

The minimum inhibitory concentrations (MIC) were determined in 96-well plates (eight rows marked from A-H and 12 columns marked from 1–12). Two-hundred microliters of each AqEO respectively MiEO were distributed in the first well of each row (A-F). One-hundred microliters of sterile distilled water were distributed in the columns 2 to 12. Using a multichannel pipette, 100 µL of AqEO / MiEO from the first column were sequentially mixed the water, achieving two-fold seriated dilutions. The remaining 100 µL from the last dilution mix were discarded. The final concentrations of essential oils were 50%, 25%, 12.5%, 6.25%, 3.13%, 1.56%, 0.78%, 0.39%, 0.20%, 0.10%, 0.05% and 0.025% v/v in final volume (or as reported by other authors and rounded: 500, 250, 125, 64, 32, 16, 8, 4, 2, 1, 0.5 and 0.25 µL/ml). Two plates were prepared for each bacterial strain (one for AqEO and one for MiEO testing).

Forty-eight hours before the study, the bacterial strains were revitalized on Columbia blood agar (Oxoid Limited, Pratteln, UK) and checked for purity. From an isolated culture, a standard inoculum of 0.5 McFarland units (2 × 10^8^ colony forming units/µL) was prepared in sterile saline. Ten microliters of inoculum were transferred in 9990 µL of 2x concentrated Mueller-Hinton broth (Oxoid Limited, Pratteln, UK), and 100 µL were transferred with a multichannel pipette from a pipetting tray over the 100 µL of AqEO/MiEO, obtaining a bacterial inoculum of approximatively 2 × 10^4^ CFU/well in a final volume of 200 µL. Negative and positive control wells were prepared for each plate, in the last row (Mueller-Hinton broth and water for negative control in well H11, respectively bacterial inoculum and water, without essential oils for positive control in well H12).

The plates were incubated at 35 °C for 18 hours in normal atmosphere. The MIC was interpreted in the last well of each row where no visible bacterial growth was noticed (bacterial growth inhibition), and interpreted as v/v percentage of stock solution.

### 2.6. Determination of Minimum Bactericidal Concentrations

The minimum bactericidal concentrations (MBC) were determined from the last three wells of each row that showed no bacterial growth after plate incubation. For this, 3 µL from the corresponding wells were spot-inoculated on blood-agar plates, which were labeled in a checkerboard pattern with the corresponding wells coordinates. The plates were incubated overnight at 35 °C, and the colony development was followed in each spot-inoculation place. The MBC was noted for the position where no bacterial colonies were developed and interpreted as v/v percentage of stock solution.

### 2.7. Interpretation of Results

The results were analyzed in spreadsheet software (Microsoft Excel 2007) and then organized in tables for better overall comparative analysis of the MIC/MBC. All the values were also normalized against the MIC/MBC values of pure ethylic alcohol (Chemical Company, Romania), as determined in previously published results of the author, using the same methodology and bacterial strains [16]. An index of 1 signifies an identical effect with the ethylic alcohol. Indexes higher than 1 or lower than 1 signify a higher respectively lower activity of the essential oil compared with ethylic alcohol. Statistical analysis was performed in GraphPad InStat 3 software (GraphPad Software, Inc., San Diego, CA, USA), with a significance level of p < 0.05 (95% confidence level). In order to be able to calculate the significance in two sample-paired *t*-tests, all MIC/MBC values that were not quantifiable (>50%) were considered as the next twofold dilution factor (100%).

## 3. Results

The chemical composition of the tested essential oils showed high percentage of β-pinene in frankincense (25.6%), myrtle (25%) and lemon (15.1%) essential oils. α-Terpinene was found in higher percentage only in frankincense (18.6%). Eucalyptol was found mainly in myrtle oil (28.7%). Linalool was found in high percentage in thyme (56.5%) and lavender (25%) oils. Thyme also contained 16.3% geranyl propionate. Limonene was found in high percentage in lemon essential oil (36.9%), aside α- and β-pinene (19.2%, respectively 15.1%). Oregano essential oil contained predominantly carvacrol (80.5%). Lavender oil contained mainly linalyl-butyrate (26.5%), linalool (25%) and other compounds with concentrations ranging between 3%–7%: geranyl butyrate, β-caryophyllene, terpinyl acetate, farnesol, ocimene, 4-carvomenthenol.

**Figure 1:**
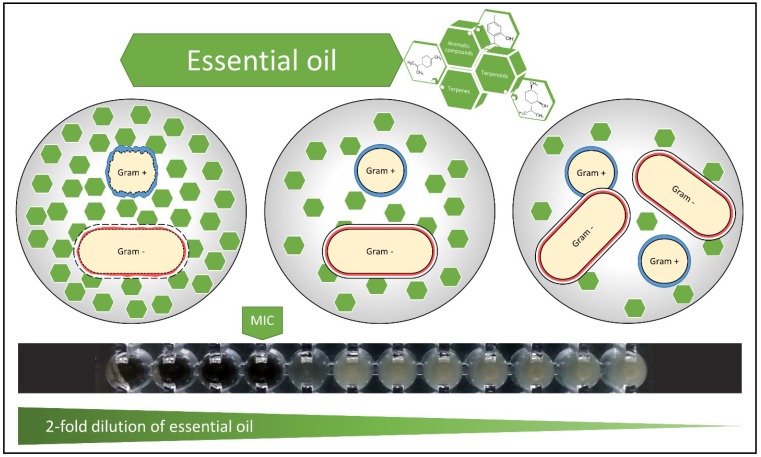
Effects of essential oil on bacteria. Gram-positive peptidoglycan cell wall allows hydrophobic molecules to penetrate and reach the internal environment. The lipopolysaccharide, which is part of the external layer of Gram-negative bacteria, allows mainly small hydrophilic molecules to pass and is only partly permissive for hydrophobic molecules. The hydrophobicity of essential oils is responsible for the disruption of bacterial structures. The mechanisms of action of essential oil on bacteria are: degradation of the cell wall and cytoplasmic membrane, cytoplasm coagulation and diffusion through the double lipid layer of the membrane, together with alteration of its permeability and function [18]. The black ribbon shows a twofold dilution set for aqueous extract of lavender oil and its effect against *Pseudomonas aeruginosa* (MIC = 6.3%; MBC was found to be >50%).

We tested the inhibitory and bactericidal effects of six essential oils (in form of aqueous extract and micelles) on six bacterial strains that are representative for the most common pathogenic species: methicillin sensitive *Staphylococcus aureus*, methicillin-resistant *Staphylococcus aureus*, *Enterococcus faecalis*, *Escherichia coli*, *Klebsiella pneumoniae* and *Pseudomonas aeruginosa*. The susceptibility of these bacterial strains to antimicrobial compounds is different, mainly due to their specific morphology (bacterial cell wall structure, capsule formation) (Figure 1).

As presented in Table 1 and Table 2, the best activity was achieved by micelles suspension of essential oils, with MICs and MBCs ranging from 0.1% to >50% v/v. The most active oils were oregano, thyme, lemon and lavender. MiEO of oregano managed to achieve bactericidal effect at concentrations as low as 0.1–6.3% v/v on all tested bacteria. The aqueous extracts showed generally lower bacteriostatic and bactericidal activity, ranging from 6.3% to >50% v/v. In one specific case, aqueous extract of lavender oil presented good bacteriostatic effect on *Pseudomonas aeruginosa* (MIC = 6.3%), better than its corresponding micellar suspension (MIC > 50%). In two other cases, also for lavender oil, the aqueous extract presented better bacteriostatic and bactericidal effect (MIC/MBC = 6.3%) than its micellar suspension (MIC/MBC = 12.5%) on *Klebsiella pneumoniae*. This can be explained by the presence of polysaccharide capsule of *Klebsiella* species, which acts like a barrier against hydrophobic oils, but allows the diffusion of aqueous extracts.

The most susceptible bacteria were the Gram-positive cocci, followed by the representatives of *Enterobacteriaceae* family. Negligible differences were noticed between MSSA and MRSA response to the essential oils; though, we have to note that MRSA was more susceptible to MiEO, and less susceptible to AqEO than MSSA. *Pseudomonas aeruginosa* presented the highest resistance against most of the essential oils, except for the oregano and lemon.

The least active oils were frankincense and myrtle, with a mild effect on Gram-positive cocci (MICs of 50% and higher). Notably, myrtle successfully inhibited *Enterococcus faecalis* at a concentration of 6.3%, but these bacteria maintained their viability even at more than 50% myrtle concentration.

Considering ethylic alcohol as a standard antimicrobial, we observed that micellar solutions, especially oregano oil present up to 64 times lower MICs/MBCs on Gram-positive cocci and on enteric Gram-negative rods. Thyme micellar oil was also found to be 2–4 times more potent than ethylic alcohol. Aqueous solutions were less effective than ethanol on all bacteria (Table 3). 

The biggest differences between MiEO and AqEO were noticed in oregano oil, regarding both MIC and MBC (*p* = 0.006). Statistical differences were observed also in case of thyme and lemon oils, where the MIC/MBC were significantly lower for micellar suspensions. No statistical differences between overall MIC/MBC of MiEO and AqEO were noticed in case of frankincense, myrtle and lavender oils (Table 4).

## 4. Discussions

Essential oils have been used since ancient times in aromatherapy and disease control, after observational effects, without knowing the substrate or the interaction with the micro- or macroorganism. Many essential oils are available nowadays, and they are widely used according to their availability and local traditions.

Frankincense is a resin of *Boswellia* tree and represent one of the most common incenses used in ceremonies and in biblical history. Nowadays it is also used in cosmetics, aromatherapy and pharmaceutical products. The composition is mostly monoterpenes (we also found β-pinene as the main component—25.6%), sesquiterpenes, monoterpenols, sesquiterpenols and boswellic acids, but very low amounts of terpenoids [19]. It was shown to have antiasthmatic and antithrombotic properties, inhibit tumor growth and induce tumor cell apoptosis [20,21].

Myrtle oil is used nowadays in contemporary Christianity as incense. In medicine, it is described to have many protective effects for several conditions, especially digestive and skin disorders [22]. It is composed of bioactive constituents such as polyphenols, terpenes (which we also found β-pinene—25%), acylphloroglucinol, myrtenyl acetate, limonene, and linalool with antioxidant and antimicrobial effects [23,24]. We also found a monoterpenoid (eucalyptol) in high percentage (28.7%). For both frankincense and myrtle, we found a very low antibacterial effect, and only on Gram-positive cocci, with high MICs. We have to note that myrtle oil presented a very good inhibitory effect on *Enterococcus* spp., which was described in other studies without a plausible explanation [25]. Metabolic differences may have a role in resistance to frankincense and myrtle, as *Enterococcus faecalis* is the single tested species without catalase production.

The lemon (*Citrus limon*) fruits are widely used mainly for culinary purposes all around the world. The major compounds of lemon essential oil were shown to be limonene (37.5%) and β-pinene (17.9%) [26], but we also found a good concentration of α-pinene (19.2%). Fewer studies on lemon oil were published, compared with other extracts. Though, it was shown that lemon oil exerts antibacterial and antioxidant properties, present cytoprotective activity and is parasitic repellant [26,27,28]. Our results also support the previous findings, micellar lemon oil presenting good MICs and MBCs on all bacteria, probably due to limonene and pinenes.

Oregano (*Origanum vulgare*) is another popular herb and originates from the Mediterranean region, but nowadays used globally as food flavoring. Oregano essential oil has been used in folk medicine since ancient times and presents antioxidant and antibacterial activity [29,30]. A main component of oregano essential oil (more than 60% of the oil composition) is carvacrol, a monoterpenoid phenol, which can be also be found in thyme or bergamot, but in lower concentrations [31]. Our oregano oil sample presented a very high content of carcavrol (80.5%), which supports the antimicrobial effect findings. Carvacrol presents a good antibacterial activity by permeabilization and depolarization of the cytoplasmic membrane, as shown in several recent studies [32,33]. Carvacrol also inhibit microbial toxin production, inhibit biofilm formation, reduce the fimbriae production and the swarming motility of uropathogenic *E. coli* and present anti-inflammatory effects [34,35]. Carvacrol is slightly soluble in water, at a concentration of 1.25 mg/mL at 25 °C [36]. The high carvacrol content of oregano oil together with its water solubility may explain the good antibacterial effect of its aqueous extract compared with the aqueous extract of other essential oils. Oregano oil presented the best antibacterial effect and was the only tested oil that presented bactericidal effect on all tested bacteria, including *Pseudomonas aeruginosa* and MRSA. Only the thyme aqueous extract presented comparable effect (though not on *Pseudomonas aeruginosa*), but the carvacrol content of thyme oil is lower than in oregano oil, of up to 45% according do other studies [37] (though in our thyme essential oil sample, carvacrol was not present). The hydroxyl group position is the single difference between carvacrol and thymol, but this, together with the terpenes’ concentrations in oils, can explain the slightly different activity. Due to this difference, carvacrol also better protects against *Pseudomonas* spp. biofilm development [38]. It was previously shown that carvacrol and thymol impairs the cell membrane, increase the membrane permeability and decrease the cytoplasmic pH in *Pseudomonas aeruginosa* and *Staphylococcus aureus* [39]. 

Thyme (*Thymus vulgaris*) is a very popular herb that plays important role in world cuisine. It is a flavoring for many meat-based foods. Its essential oil was shown to have antimicrobial, and anti-inflammatory properties [40]. According to our results, the thyme micelles and aqueous extract presented comparable effect with oregano oil, occupying the second place for antibacterial effect. According to some studies, thyme oil presents almost half of its content thymol (46%), followed by lower amounts of γ-terpinene, carvacrol and p-Cymene [41]. Essential oils of thyme species that contain higher amounts of carvacrol and thymol, present stronger antimicrobial effect [42]. In our thyme oil sample, more than half of its content was linalool (56.5%). Linalool is soluble in water at a concentration of 1.59 mg/mL at 25 °C [43], which can explain the good aqueous extract activity of thyme. *Pseudomonas aeruginosa* was found highly resistant to other essential oils, derived from green leaves plants (parsley, thyme, basil), but thyme expressed strong effect on Gram-negative enteric rods [44].

Lavender (*Lavandula angustifolia*) flowers are usually used for decoration and fresh fragrance. Lavender essential oil is used in cosmetics, and not much about antimicrobial effects was published. Its main constituents are linalool and linalyl-acetate which sum more than 50%, followed by smaller amounts of sesquiterpenes and terpenes [45]. The high content of linalyl-butyrate we found (26.5%) explains the good antibacterial activity of lavender micellar form, while the linalool (25%) and its solubility in water may explain the good inhibitory effect of its aqueous extract. 

Some big differences were found between the MIC and MBC of the same sample. For example, lavender oil presents generally very low MICs, but very high MBCs. This can be explained because some components that alter the cell division. For example, cinnamaldehyde was found to inhibit FtsZ protein assembly into a Z-ring; or many proteins are upregulated or downregulated by the presence of thymol; carvacrol affect the proteins involved in cell division [18].

A PubMed search for the terms [“essential oil” AND (antimicrobial OR antibacterial)] provided more than 3000 results, with an ascending trend from 2000, about half of these dating from 2013. The interest for alternative to antibiotics is necessary, as the antibiotic era will soon end, according to specialists’ opinions. The essential oils, as natural extracts, with low adverse effects, may become reliable alternatives in antimicrobial fight. Many patients could be of benefit to local treatments with essential oils. Use of these alternative topic treatments could be useful in conditions where the bacterial colonization/infection are hard to control, such as necrotic ulcers, diabetic feet, or where the blood flow is reduced, or other situations where the oral or intravenous antibiotics do not reach the infection site. Also, the fight against multidrug resistant strains has to be won by other means than antibiotics, and natural extracts could be part of this. Further studies are needed in order to find the proper ways to deliver the active antimicrobial compounds.

The present results suggest that some forms of colloid or micelle suspension of whole essential oils may assist in antimicrobial fight, especially those that contain high amounts of terpenes and terpenoids (such as oregano, thyme or lemon oil) which are active against many bacterial species, including highly resistant and adaptable ones such as MRSA or *Pseudomonas* spp. In selected cases, creams that can penetrate the corneous skin layer may contain aqueous extracts of oregano or thyme oil, with good antibacterial activity. So, based on the most recent literature, we can hypothesize the possible application of new therapeutic protocols for resistant infectious diseases, based on the use of essential oils and probiotics [30,35,46,47,48,49].

## Figures and Tables

**Table 1:** MICs of micellar and aqueous extracts of the six essential oils.

		Frankincense	Myrtle	Thyme	Lemon	Oregano	Lavender
MiEO	MSSA	50%	50%	3.1%	6.3%	0.4%	3.1%
	MRSA	50%	50%	3.1%	6.3%	0.2%	3.1%
	*E. faecalis*	50%	6.3%	6.3%	12.5%	0.8%	6.3%
	*E. coli*	>50%	>50%	1.6%	6.3%	0.1%	6.3%
	*K. pneumoniae*	>50%	>50%	3.1%	12.5%	0.2%	12.5%
	*P. aeruginosa*	>50%	>50%	50%	12.5%	6.3%	>50%
AqEO	MSSA	>50%	>50%	12.5%	25%	12.5%	25%
	MRSA	>50%	>50%	25%	50%	12.5%	50%
	*E. faecalis*	>50%	>50%	12.5%	50%	25%	12.5%
	*E. coli*	>50%	>50%	12.5%	50%	12.5%	12.5%
	*K. pneumoniae*	>50%	>50%	12.5%	25%	6.3%	6.3%
	*P. aeruginosa*	>50%	>50%	>50%	>50%	12.5%	6.3%

**Table 2:** MBCs of micellar and aqueous extracts of the six essential oils.

		Frankincense	Myrtle	Thyme	Lemon	Oregano	Lavender
MiEO	MSSA	>50%	>50%	12.5%	25%	1.6%	>50%
	MRSA	>50%	50%	6.3%	6.3%	0.8%	>50%
	*E. faecalis*	>50%	>50%	12.5%	50%	1.6%	>50%
	*E. coli*	>50%	>50%	3.1%	12.5%	0.1%	6.3%
	*K. pneumoniae*	>50%	>50%	3.1%	12.5%	0.2%	12.5%
	*P. aeruginosa*	>50%	>50%	50%	25%	6.3%	>50%
AqEO	MSSA	>50%	>50%	12.5%	>50%	25%	>50%
	MRSA	>50%	>50%	50%	>50%	50%	>50%
	*E. faecalis*	>50%	>50%	50%	>50%	25%	>50%
	*E. coli*	>50%	>50%	12.5%	>50%	50%	25%
	*K. pneumoniae*	>50%	>50%	12.5%	25%	6.3%	6.3%
	*P. aeruginosa*	>50%	>50%	>50%	>50%	50%	>50%

**Table 3:** Normalized index values of MICs and MBCs of micellar and aqueous extracts of the six essential oils, compared to the effect of pure ethylic alcohol.

		Frankincense		Myrtle		Thyme		Lemon		Oregano		Lavender	
		MIC	MBC	MIC	MBC	MIC	MBC	MIC	MBC	MIC	MBC	MIC	MBC
MiEO	MSSA	0.13	0.25	0.13	0.25	**2**	**2**	1	1	**16**	**16**	**2**	0.25
	MRSA	0.13	0.13	0.13	0.25	**2**	**2**	1	**2**	**32**	**16**	**2**	0.13
	*E. faecalis*	0.13	0.25	1	0.25	1	**2**	0.50	0.50	**8**	**16**	1	0.25
	*E. coli*	0.06	0.06	0.06	0.06	**4**	**2**	1	0.50	**64**	**64**	1	1
	*K. pneumoniae*	0.03	0.06	0.03	0.06	1	**2**	0.25	0.50	**16**	**32**	0.25	0.50
	*P. aeruginosa*	0.02	0.13	0.02	0.13	0.03	0.25	0.12	0.50	0.25	**2**	0.02	0.13
AqEO	MSSA	0.06	0.25	0.06	0.25	0.50	**2**	0.25	0.25	0.50	1	0.25	0.25
	MRSA	0.06	0.13	0.06	0.13	0.25	0.25	0.13	0.13	0.50	0.25	0.13	0.13
	*E. faecalis*	0.06	0.25	0.06	0.25	0.50	0.50	0.13	0.25	0.25	1	0.50	0.25
	*E. coli*	0.06	0.06	0.06	0.06	0.50	0.50	0.13	0.06	0.50	0.13	0.50	0.25
	*K. pneumoniae*	0.03	0.06	0.03	0.06	0.25	0.50	0.12	0.25	0.50	1	0.50	1
	*P. aeruginosa*	0.02	0.13	0.02	0.13	0.02	0.13	0.02	0.13	0.12	0.25	0.25	0.13

**Table 4:** Differences between the average MICs/MBCs of micellar and aqueous extracts of the tested essential oils. The significant results are highlighted. Thyme, lemon and oregano oils present significantly lower overall MICs and MBCs in their micellar form than their aqueous extracts.

	MIC					MBC				
	MiEO		AqEO		*p*-Value	MiEO		AqEO		*p*-Value
	Mean	SD	Mean	SD		Mean	SD	Mean	SD	
Frankincense	75%	27.4%	100%	0%	0.076	100%	0%	100%	0%	N/A
Myrtle	67.7%	38.8%	100%	0%	0.097	91.7%	20.4%	100%	0%	0.363
Thyme	**11.2%**	19.1%	29.2%	35.1%	**0.045**	**14.6%**	17.9%	39.6%	34.8%	**0.034**
Lemon	**9.4%**	3.4%	50%	27.4%	**0.013**	**21.9%**	15.7%	87.5%	30.6%	**0.003**
Oregano	**1.3%**	2.4%	13.5%	6.1%	**0.006**	**1.7%**	2.3%	34.4%	18.4%	**0.006**
Lavender	21.9%	38.4%	18.8%	16.8%	0.879	69.8%	46.8%	71.9%	44%	0.576
