# Erratum: Man, A., et al. Antimicrobial Activity of Six Essential Oils Against a Group of Human Pathogens: A Comparative Study. *Pathogens* 2019, *8*, 15

**DOI:** 10.3390/pathogens8030108

**Published:** 2019-07-25

**Authors:** Adrian Man, Luigi Santacroce, Romeo Iacob, Anca Mare, Lidia Man

**Affiliations:** 1 Department of Microbiology, University of Medicine, Pharmacy, Sciences and Technology of Târgu Mureș, Târgu Mureș 540139, Romania; 2 Ionian Dept. (DJSGEM), Microbiology and Virology Lab., University Hospital of Bari, 70124 Bari, Italy; 3 Polypheno srl Academic Spin Off, University of Bari, 70124 Bari, Italy; 4 University of Medicine, Pharmacy, Sciences and Technology of Târgu Mureș, Târgu Mureș 540139, Romania; 5 Pediatrics Clinic 1, University of Medicine, Pharmacy, Sciences and Technology of Târgu Mureș, Târgu Mureș 540139, Romania

The authors would like to make the following corrections to the published paper [1]. The changes are as follows:

(1) The author’s name should be corrected as follows: 

Romeo Jacob

with 

Romeo Iacob

(2) The email address of Adrian Man should be corrected as follows:

adrian.man@umftgm.ro

with

adrian.man@umfst.ro

The authors and the Editorial Office would like to apologize for any inconvenience caused to the readers by these changes. The change does not affect the scientific results. The manuscript will be updated and the original will remain online on the article webpage.
